# The Children’s Attention Project: a community-based longitudinal study of children with ADHD and non-ADHD controls

**DOI:** 10.1186/1471-244X-13-18

**Published:** 2013-01-10

**Authors:** Emma Sciberras, Daryl Efron, Elizabeth J Schilpzand, Vicki Anderson, Brad Jongeling, Philip Hazell, Obioha C Ukoumunne, Jan M Nicholson

**Affiliations:** 1 Murdoch Childrens Research Institute, Melbourne, Australia; 2 Centre for Community Child Health, The Royal Children’s Hospital, Melbourne, Australia; 3 Parenting Research Centre, Melbourne, Australia; 4 Department of Psychological Sciences, University of Melbourne, Melbourne, Australia; 5 Integrated Mental Health Program, The Royal Children’s Hospital, Parkville, Australia; 6 Joondalup Child Dev Centre, Perth, Australia; 7 Department of Paediatrics, University of Western Australia, Perth, Australia; 8 Discipline of Psychiatry, University of Sydney, Sydney, Australia; 9 PenCLAHRC, Peninsula College of Medicine and Dentistry, University of Exeter, Exeter, UK; 10 Department of Paediatrics, University of Melbourne, Melbourne, Australia; 11 Murdoch Childrens Research Institute, The Royal Children’s Hospital, Flemington Road, Parkville, Vic, 3052, Australia

**Keywords:** Attention deficit disorder with hyperactivity, Child, Longitudinal studies, Community, Outcome assessment

## Abstract

**Background:**

Attention-Deficit/Hyperactivity Disorder (ADHD) affects approximately 5% of children worldwide and results in significant impairments in daily functioning. Few community-ascertained samples of children with ADHD have been studied prospectively to identify factors associated with differential outcomes. The Children’s Attention Project is the first such study in Australia, examining the mental health, social, academic and quality of life outcomes for children with diagnostically-confirmed ADHD compared to non-ADHD controls. The study aims to map the course of ADHD symptoms over time and to identify risk and protective factors associated with differential outcomes.

**Methods/design:**

The sample for this prospective longitudinal study is being recruited across 43 socio-economically diverse primary schools across Melbourne, Australia. All children in Grade 1, the second year of formal schooling (6–8 years), are screened for ADHD symptoms using independent parent and teacher reports on the Conners’ 3 ADHD index (~N = 5260). Children screening positive for ADHD by both parent and teacher report, and a matched sample (gender, school) screening negative, are invited to participate in the longitudinal study. At baseline this involves parent completion of the NIMH Diagnostic Interview Schedule for Children IV (DISC-IV) to confirm likely ADHD diagnostic status and identify other mental health difficulties, direct child assessments (cognitive, academic, language and executive functioning; height and weight) and questionnaires for parents and teachers assessing outcomes, as well as a broad range of risk and protective factors (child, parent/family, teacher/school, and socio-economic factors). Families will be initially followed up for 3 years.

**Discussion:**

This study is the first Australian longitudinal study of children with ADHD and one of the first community-based longitudinal studies of diagnostically confirmed children with ADHD. The study’s examination of a broad range of risk and protective factors and ADHD-related outcomes has the potential to inform novel strategies for intervention and prevention.

## Background

Attention-Deficit/Hyperactivity Disorder (ADHD) is the most common neurodevelopmental disorder, affecting approximately 5% of children worldwide [1]. It is now the most common reason for paediatrician presentations in Australia, accounting for 18% of general consultations [2]. Children with ADHD exhibit developmentally inappropriate levels of inattention, hyperactivity and/or impulsivity which result in a range of impairments in social, educational, and family functioning [3]. ADHD can be categorised according to three subtypes: ADHD combined type (ADHD-C); ADHD predominantly inattentive type (ADHD-I); and ADHD predominantly hyperactive type (ADHD-H). Although ADHD-C is the most common diagnosis made in clinical settings, ADHD-I accounts for approximately 50% of all identified cases within community-ascertained samples [4].

ADHD is characterised by deficits in executive functioning (e.g., poor working memory; impaired planning and sustained attention) [5] and motivation (e.g., altered processing of reinforcement and incentives) [6], which appear to be underpinned by disordered fronto-striato-cerebellar brain circuitry [7]. ADHD is the result of complex causal pathways involving interactions between a range of genetic and environmental factors [8]. Symptoms are typically evident in early childhood, attracting increased parental and professional concern when children enter structured education settings, with diagnosis commonly made in the primary school years [4].

### Outcomes for children with ADHD

There is a substantial literature addressing the aetiology and treatment of ADHD, with studies examining the course of ADHD over time mostly focussed on children selected through clinical settings [9-13]. These studies of clinical samples have demonstrated that both boys and girls with ADHD are at risk for a range of poorer outcomes in adolescence and adulthood. For example, in terms of mental health, children with ADHD have higher rates of externalising behaviour disorders (e.g., oppositional defiant disorder, conduct disorder) than non-ADHD controls [14,15], with mixed evidence regarding risks for substance use and mood and anxiety disorders [13,16-18]. Children with ADHD also have increased risk for poor educational (e.g., grade repetition, suspensions, lower attainment and achievement), social (e.g., fewer friends, trouble maintaining friendships) [13,14,19], and occupational outcomes (e.g., poorer job performance, higher employer-rated ADHD symptoms) [14]. The parents of children with ADHD also have poorer outcomes over time, including increased psychological distress and poorer family functioning than the parents of non-ADHD controls [9]. ADHD symptoms persist into adolescence and adulthood for approximately 50% of children diagnosed with ADHD in childhood [20]. However, apart from evaluations of specific medication, behavioural or educational interventions, little is known about the factors associated with symptom remission. Although there may be a decline in ADHD symptoms as children progress into adolescence and adulthood, the impairment associated with the disorder tend to persist [20].

While previous research provides valuable information about the outcomes for clinic-referred children with ADHD, it tells us little about children not accessing clinical services [21,22]. Australian population studies show cross-sectionally that as few as 1 in 5 children and adolescents with mental health difficulties had accessed professional services within the six months prior to being surveyed [23]. Compared with children with ADHD in the general population, clinical samples over-represent children with more severe ADHD, males, and those with comorbid mental health conditions [21,22]. In turn, clinical samples under-represent those with ADHD-I and females with ADHD [21,22].

### Community-based longitudinal studies of children with ADHD

Community-based longitudinal designs have the potential to address knowledge gaps regarding the course of ADHD and provide findings that are more applicable to the general population of children with ADHD. However, most existing community-based studies are limited in their assessment of ADHD status. For example, one common approach is to ask parents whether their child has ever received a diagnosis of ADHD. This approach is likely to contain measurement error arising from parents who indicate ‘yes’ based, for example, on professional opinion rather than formal diagnostic assessment. Furthermore, this approach does not assess whether symptoms are current, and as with the studies of clinical samples of children with ADHD, this approach will only include children who do get referred and assessed for ADHD. The second common approach is to define ADHD status using parent and/or teacher-reported ADHD symptom checklists [24,25]. The limitation of this approach is that it does not assess two key aspects required for ADHD diagnosis – namely, the persistence of symptoms for a period of at least six months, and significant functional impairment.

To date, there have been few community-based longitudinal studies of children with diagnostically confirmed ADHD and controls [26-29]. Barberesi and colleagues report on the outcomes of a population-based birth cohort of ADHD incident cases (n = 379) and non-ADHD controls (n = 758) in Rochester, Minnesota [26,29]. Children with ADHD were found to have poorer educational and psychiatric outcomes than non-ADHD controls [26,29]. However, the study employed a retrospective design and direct assessment of ADHD, mental health disorders and educational outcomes was not possible. Rasmussen and Gillberg [28] followed a Sweedish community sample of children with ADHD (combined with Developmental Coordination Disorder) into adulthood. Their sample of children with ADHD-only was too small at follow-up for meaningful analyses (n = 11). Bussing and colleagues report 8 year outcomes for 5 to 11 year old North American school-children with ADHD (n = 95), subthreshold ADHD (n = 75), and a comparison group (n = 163) [27]. At follow-up, children with ADHD had poorer mental health, quality of life, and increased contact with the juvenile justice system compared with non-ADHD controls. The wide age range at baseline and the large gap between baseline and outcome assessment precluded detailed examination of how children’s symptom trajectories changed over time.

Further research in this area would benefit from prospective community-based designs that include both direct assessment of children’s functioning and multi-source (parent, teacher, child) data on potential risk and protective factors for poor long-term outcomes. Regular follow-up assessments are required to enable examination of subtle changes in children’s symptoms. This is important in understanding the developmental trajectories for children with ADHD and the predictors of poor versus better outcomes in this population.

### Predictors of outcomes for children with ADHD

**Figure 1:**
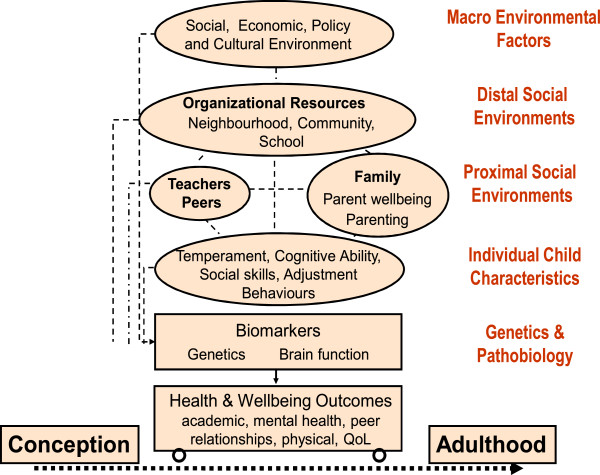
Socio-ecological model underpinning the Children’s Attention Project design.

Socio-ecological models that highlight the multitude of individual and contextual factors shaping children’s health and development provide a useful framework for conceptualising the factors likely to influence the persistence of ADHD and associated outcomes. The socio-ecological model of children’s health that underpins the selection of constructs to be measured in our study is shown in Figure 1.

Macro-environmental (e.g., social, economic, policy factors) and distal social environments (e.g., neighbourhood, school, community factors) are important factors influencing children’s developmental trajectories, but little attention has been given to these factors within existing longitudinal studies examining outcomes for children with diagnostically-confirmed ADHD. Key proximal influences are also critical and may include parent–child interactions, parental mental health, and children’s relationships with their teachers and peers. Longitudinal studies of children with ADHD have identified that family context [30-32], child factors (e.g., IQ, comorbid mental health problems; ADHD severity/subtype) [15,31,33,34], homework management [35] and interventions (education programs; stimulant medication) [36] may influence outcomes. However, findings have been mixed and studies have yet to examine a comprehensive range of predictors within the one study. Furthermore, the existing research examining predictors of outcomes is largely characterised by the sampling limitations noted for previous cohort studies, and to date there has been little attention to the potential risk or protective roles of a broad range of early school factors such as child-teacher relationships.

### Study aims

The present study will establish a community-based cohort of 200 6–8 year old children with diagnostically-confirmed ADHD and matched non-ADHD controls. The cohort will be tracked across the early years of primary (elementary) school to determine the mental health, academic and social outcomes for children with and without ADHD and to identify the factors associated with differential outcomes within the ADHD group. Within a community-based sample, the primary aims of this study are to:

1. measure ADHD symptoms over the early school years;

2. evaluate the extent to which children with ADHD have elevated risks for poor academic, mental health, quality of life, and social outcomes compared to non-ADHD controls;

3. evaluate the extent to which parents of children with ADHD have elevated risks for poor mental health and quality of life compared to non-ADHD controls; and

4. assess the influence of ADHD subtype and symptom severity, comorbidities, and other child, family, socioeconomic and school factors on mental health, academic, quality of life, and social outcomes.

## Methods/design

### Overall study design

**Figure 2:**
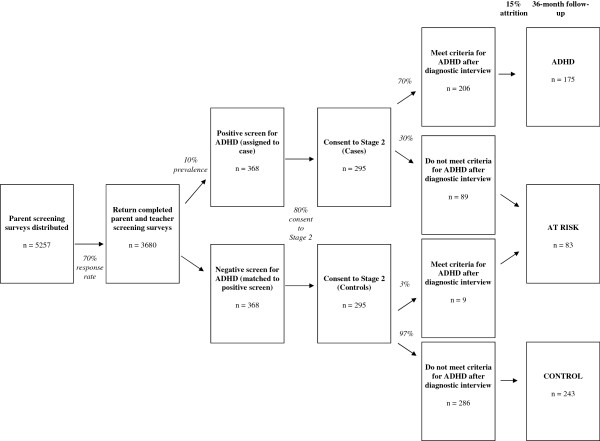
Children’s Attention Project study design.

The Children’s Attention Project is a longitudinal cohort study conducted in accordance with STROBE guidelines [37]. The project commenced in early 2011, with the cohort to be screened and recruited from two consecutive years of Grade 1 enrolments (cohort 1 in 2011, cohort 2 in 2012). Baseline, 12, 24 and 36 month follow-ups will be conducted across 2011 to 2015. The anticipated flow of participants for the two cohorts combined is shown in Figure 2, based on data from a pilot study (n = 345). The study has been funded by the National Health and Medical Research Council of Australia (project grant number: 1008522) and internal funding from the Murdoch Childrens Research Institute. It has been granted approval by the Human Research Ethics Committees of the Royal Children’s Hospital (#31056) and the Victorian Department of Education and Early Childhood Development (#2011_001095).

### Participants

Participants are Grade 1 (which is the second year of formal schooling in Victoria) children and their parents and teachers from 43 government primary schools in Melbourne, Victoria. Grade 1 was chosen as the recruitment point, as this is a developmental stage at which children with ADHD often begin to manifest impairments related to the functional impact of their symptoms. Earlier sampling would result in missed cases. Although later sampling would increase the yield of cases, opportunities to reliably record and then track key early influences on developmental trajectories would be lost.

Exclusion criteria include: intellectual disability; serious medical conditions; genetic disorders; moderate-severe sensory impairment; neurological problems; and parents with insufficient English to complete interviews/questionnaires.

### Procedure

#### School recruitment

Schools were recruited via the state department responsible for government schools (Victorian Government Department of Education and Early Child Dev). Five school networks in two Melbourne metropolitan regions (Eastern and Western) were approached. The networks were selected to provide access to schools from diverse socioeconomic communities as recorded in administrative departmental data using the Index of Community Socioeconomic Advantage (ICSEA), which has a mean of 1000 and a standard deviation of 100 [38]. Following presentations at regional network meetings, school principals who indicated an interest in the study were visited and meetings arranged with teachers to discuss the project details. Schools were accepted into the study if teachers indicated support and principals signed a research agreement regarding the school’s contribution to data collection. To facilitate data collection, teacher relief funding is provided to schools to compensate for teacher participation in data collection and a school prize is provided each year to the school in each region with the highest proportion of parent consent forms returned (both accepted and declined consent counted).

#### Initial screening for ADHD and non-ADHD sample

With parental consent, all Grade 1 children in 2011 and 2012 in participating schools are screened for ADHD symptoms using a two-step process. The first step involves parent completion of two-page screener containing: the 10-item Conners 3 ADHD Index [39]; a question asking whether the child has been previously diagnosed with ADHD; questions assessing exclusion criteria; brief demographic details (e.g., child date of birth and gender; language spoken at home); and contact details if parents consent to subsequent follow-up (outlined below). Parent screeners and information sheets are sent home with children and returned in sealed envelopes via classroom collection boxes or reply paid mail. Reminder letters and replacement screeners are sent home to those who do not decline and have not returned the screener at two and four weeks after the initial distribution. The second step of screening involves teacher completion of the Conners 3 ADHD Index for all children whose parents provide consent. Reminders and replacements are sent after two weeks, with school-nominated liaison personnel following up on outstanding teacher screeners as required.

Children are considered to have screened positive for ADHD if scores on *both* parent and teacher Conners 3 ADHD Indices are equal to or above a percentile cut-point for age and gender (high score = high indicating more symptoms). The 75^th^ percentile is used for boys and the 80^th^ percentile for girls. These cut-points were selected based on our pilot study data, which demonstrated good correspondence with DSM-IV ADHD criteria on the NIMH Diagnostic Interview Schedule for Children IV (DISC-IV) [40]. As cross-situational impairment is one of the key requirements for a diagnosis of ADHD, we require children to have elevated symptoms by both parent and teacher report to be considered to have screened positive. Although the DISC-IV asks about impairment in the school context due to ADHD symptoms, this is by parent report only, therefore having corroborating teacher report data provides the necessary independent information that the child is symptomatic at school, as well as at home. Additionally, any child reported by parents as already having received a diagnosis of ADHD is automatically regarded as a positive screen. The parents of all children who screen positive, who do not meet exclusion criteria and where consent for the next stage has been provided, are contacted and invited to participate in the longitudinal study which involves diagnostic confirmation and baseline assessment.

Children are considered to have screened negative for ADHD and eligible for inclusion if scores on *both* parent and teacher Conners 3 ADHD Indices are less than the 75^th^ percentile for age for boys and less than the 80^th^ percentile for age for girls, the child has no parent-reported diagnosis of ADHD, the child does not meet exclusion criteria and the parent has provided consent for further follow-up. For each positively screened child, a negatively screened child is randomly selected from the pool of negatively screened children, matched on gender and school.

#### Diagnostic confirmation and baseline data collection

The parents of all children screening positive and the randomly matched children screening negative, are telephoned to invite them into the longitudinal study. If consent is provided, all children have their ADHD status confirmed by the DISC-IV interview conducted with parents at home or at the child’s school according to preference. Direct child assessments of cognitive, academic, language, and executive functioning, plus height and weight measurements, are conducted at the child’s school. Interviews and direct assessments are completed by field staff who have at least a four-year undergraduate degree in psychology, are comprehensively trained in all procedures, and are blind to child screening status. To ensure blinding, no staff member undertakes both parent and child assessments for any given family.

With regard to medication, children are assessed in their usual classroom condition. For example, if children usually take medication for ADHD or any other mental health condition, they are not asked to cease medication for the assessment. Parents who report at the recruitment phone call that their child is taking medication to assist with emotions, learning and/or behaviour, are called the day after the child assessment to assess whether the child took any medication on the day of the assessment, and to collect additional data on the type, dose, and frequency of medication use, perceived benefits and side effects of medication.

Parents and teachers are asked to complete detailed baseline questionnaires covering a range of predictor and outcomes variables. Teachers are not informed of the screening status and diagnostic status of participating children. Parents and teachers who do not return questionnaires within two weeks, receive a reminder letter, SMS reminder and telephone call at regular intervals.

We also seek optional parent consent to retrieve/link data from complimentary data sources about health service use (Medicare and Pharmaceutical Benefit Scheme), pre-school commencement health and well-being (School Entrance Health Questionnaire), and school functioning (Australian Early Development Index), and academic functioning (National Assessment Program – Literacy and Numeracy).

#### Longitudinal follow-ups at 12, 24, and 36 months

Three groups of children will be followed up over time:

1) ADHD group: children who screen positive for ADHD and meet diagnostic criteria for ADHD on the DISC-IV;

2) At risk group: children who screen positive for ADHD but do not meet criteria for ADHD on the DISC-IV or children who screen negative for ADHD but meet criteria for ADHD on the DISC-IV; and

3) Non-ADHD controls: children who screen negative for ADHD and do not meet criteria for ADHD on the DISC-IV.

At each follow-up, parents and teachers are asked to complete detailed questionnaires with the 36 month follow-up enabling tracking of outcomes through to the cusp of puberty (age 9–11 years), a critical developmental transition point. We will use the same reminder processes as used at baseline for our parent and teacher follow-up questionnaires.

### Measures

Measures are summarised in Table 1. Parent and teacher reported measures will be collected from baseline (T1) to 36 month follow-up (T4). Parent interviews and direct child assessments collected at T1 will be repeated at T4. Measures of child outcomes are all reliable, valid scales, with excellent psychometric properties, sensitivity to intervention and widely used in Australia and internationally. Measures of possible determinants also have robust psychometric properties, are brief, and are mostly drawn from the Longitudinal Study of Australian Children (LSAC) [41,42]. Family demographic information collected at baseline (and repeated for those variables that can change over time) include parents’ age, employment, education, mental health diagnoses (including family history of ADHD), income, family composition and whether the child has ever lived for an extended period of time out of parental care. Items (mostly from LSAC) are also collected on school absences, and child and parent sleep quality. School demographics are sourced from administrative data.

**Table 1:** Summary of the measures included in the Children’s Attention Project

**Construct**	**Measures**	**Source**^a^	**Time Point**			
			**1**	**2**	**3**	**4**
**ADHD and comorbid disorders**						
ADHD symptoms	Conners’ 3 Parent & Teacher ADHD index: 10-items assessing ADHD symptom severity [39].	P,T	●	●	●	●
ADHD and comorbid disorders	DISC-IV: structured clinical interview assessing all childhood DSM-IV diagnoses [40].	P	●			●
Autism Spectrum Disorders	Social Communication Questionnaire (SCQ) – Lifetime version: 40 item measuring autism spectrum disorder symptoms [43]; Social Skills Improvement System (SSIS) – 7 item Autism Spectrum scale [44].	P	●			●
**Child, family and school determinants**						
Prenatal & postnatal factors	LSAC^b^ questions assessing alcohol use/smoking during pregnancy, gestational diabetes, preeclampsia, stress/anxiety/depression/stressful life events during pregnancy, birth weight, gestation, intensive care following birth, post-natal depression.	P	●			
Child cognitive functioning	Wechsler Abbreviated Scales of Intelligence (WASI): vocabulary, matrix reasoning [45].	C	●			●
Language	Clinical Evaluation of Language Fundamentals – 4^th^ Edition, Screening Test [46].	C	●			●
Attention skills	Test of Everyday Attention for Children (TEA-Ch): Score (auditory sustained attention, Walk, Don’t Walk (sustained attention, response inhibition)) [47].	C	●			●
Visual perception and motor coordination	Beery-Buktenica Developmental Test of Visual-Motor Integration, 6^th^ Edition (Beery VMI) [48].	C	●			●
Working memory	Wechsler Intelligence Scale for Children 4^th^ Edition (WISC IV): Digits forward, Digits backwards [49].	C	●			●
Social skills	Social Skills Improvement System (SSIS): 6 scales assessing Responsibility (6 items), Self-control (7 items), Bullying (5 items), Communication (7 items), and Engagement (7 items) [44].	P, T	●	●	●	●
Prosocial behaviour	Strengths and Difficulties Questionnaire (SDQ); 5 items assessing prosocial behaviour [50].	P, T	●	●	●	●
Parent mental health	Kessler 6 (K6): 6-item screener for psychosocial symptoms [51].	P	●	●	●	●
Family quality of life	Child Health Questionnaire (CHQ): 10-items assessing family quality of life [52].	P	●	●	●	●
Parenting	LSAC parenting scales: 20 items assessing parental warmth, inductive reasoning, hostility and consistency, as well as parental self-efficacy [53].	P	●	●	●	●
Couple relationship	LSAC family functioning scales: 20 items assessing parental conflict and support and relationship satisfaction [53].	P	●	●	●	●
Family adversity	Stressful Life Events Scale: 12 items assessing a range of stressful life events experienced in the last 12 months [54].	P	●	●	●	●
Teacher-child relationship	Student-Teacher Relationship Scale (STRS, short form): 15 items, assessing teacher-child conflict and closeness [55].	T	●	●	●	●
Teacher characteristics	Including teacher age, gender, teaching experience, education, self-efficacy, from LSAC; level of support [42].	T	●	●	●	●
**Child outcomes**						
Academic achievement	Wide Range Achievement Test (WRAT 4): word reading, numeracy [56].	C	●			●
Classroom performance	Social Skills Improvement System (SSIS): 7-item Academic Competence scale assessing classroom performance [44].	T	●	●	●	●
Physical/health	Weight and height measurement. Questions assessing child global health and sustained injuries.	C, P	●			●
Mental health and social functioning	SDQ: Total Problems score and 4 subscales (each 5 items): Emotional, Conduct, Peer, and Inattention-Hyperactivity problems. Impairment measured using the Impact scale [50]. The SSIS will also be examined as an outcome.	P, T	●	●	●	●
Quality of Life	Pediatric Quality of Life Inventory – Version 4.0 (Peds QL): 23-items assessing physical, emotional, social and school quality of life [57].	P	●	●	●	●
**Treatment and Interventions**						
Education	Questions measuring specialised school services, individual education plan, in-class assistance, and grade repetition.	T	●	●	●	●
Health and allied health	Treatment history including medication (type, dose, duration, side-effects), psychological, educational, complimentary and alternative therapies	P	●	●	●	●

^a^ P = Parent-reported measures, T = Teacher-reported measures, C = Direct child assessments.

^b^ LSAC = Longitudinal Study of Australian Children.

### Sample size

In order to detect a 0.3 SD clinically meaningful difference between the ADHD group and controls on each of our continuous primary outcomes, with 80% power and at a significance level of 0.05, we need to recruit 200 children in the ADHD group and at least 200 children in the control group. Based on data from the pilot study, we need to distribute ADHD screening questionnaires to approximately 5,300 parents in order to have data on 175 children with ADHD at follow-up. This assumes an initial 70% response rate to the combined parent/teacher screening questionnaires of whom 10% screen positive for ADHD, and an 80% consent rate to the diagnostic confirmation and baseline assessment, of whom 70% of children screening positive then meet criteria for ADHD on the DISC-IV. We have allowed for 15% attrition over the three subsequent follow-ups.

Children whose screening status differs from their subsequent diagnostic status will be followed as an ‘at risk’ sample. Based on data from our pilot study, we anticipate that approximately 30% of the sample who screen positive for ADHD and 3% of those who screen negative, will fall into the at risk group. Based on the sample size in the present study this will equate to approximately 98 children at baseline.

### Data analysis

We will compare the proportions of children meeting diagnostic criteria for other DSM-IV mental health diagnoses (DISC-IV) between the ADHD and control groups using the Chi-squared test for each of the diagnostic categories and the Mann Whitney test to compare symptom counts (oppositional defiant disorder/conduct disorder, mood and anxiety disorders, autism spectrum disorder). We will compare mean differences in parent and teacher reported total behavioural and emotional problems, social functioning, parent-reported child quality of life, measured academic achievement, and teacher-reported classroom performance between the two groups using t tests (or Mann–Whitney tests, depending on the data distribution). Furthermore, we will compare mean differences in parents’ levels of depression and family quality of life between the two groups using t tests.

We will then conduct regression analyses (linear regression for continuous data and logistic regression for binary data) for each outcome, adjusted for potential confounders identified a priori (e.g.,. child gender, family income, family structure, mother’s age, comorbidity). We will also repeat all of the above to explore differences between ADHD, at risk, and control groups.

We will conduct multivariable regression analyses (linear or logistic as appropriate) within the ADHD, at risk and control groups separately to determine the predictors of poorer (or better) functioning in each of our primary outcome domains (academic achievement, mental health problems, quality of life and social functioning). Predictors will include (depending on the outcome domain being examined): ADHD subtype, ADHD symptom severity, comorbidities, demographics, cognitive functioning, executive functioning, communication skills, parenting and couple factors, parental mental health, family QoL, teacher-child relationships, teacher/school characteristics and treatment factors. Tests of interaction between ADHD status and these predictors will be used to identify those that uniquely predict poorer or better outcomes within the ADHD group.

## Discussion

ADHD is a highly prevalent condition which results in significant impairments across multiple domains. The majority of the research examining outcomes for children with ADHD has been based on clinical samples of children with ADHD. This study will be one of the first detailed community-based longitudinal studies worldwide focusing not only on the outcomes for children with ADHD, but the predictors of outcomes for this group. By identifying the factors influencing outcomes, this study has the potential to inform novel strategies for intervention and prevention, addressing critical questions of when to intervene, with whom, and which modifiable risk and protective factors should be targeted. We are currently planning a number of additions to this project including a neuroimaging and genetic arm. By improving knowledge about the natural course of ADHD, findings will be relevant to patients and families, mental health, education, allied health and medical professionals, and policy makers. The study will also establish the foundations for subsequent data collection into late primary school, and across the transitions to adolescence and adulthood.

## Abbreviations

ADHD: Attention-Deficit/Hyperactivity Disorder; DISC-IV: NIMH Diagnostic Interview Schedule for Children IV; ICSEA: Index of Community Socioeconomic Advantage; LSAC: Longitudinal Study of Australian Children; SSIS: Social Skills Improvement System; PedsQL: Pediatric Quality of Life Inventory 4.0; WRAT 4: Wide Range Achievement Test 4; K 6: Kessler 6; CHQ: Child Health Questionnaire; SCQ: Social Communication Questionnaire; WASI: Wechsler Abbreviated Scales of Intelligence; TEA-Ch: Test of Everyday Attention for Children; Beery VMI: Beery-Buktenica Developmental Test of Visual-Motor Integration, 6^th^ Edition; WISC IV: Wechsler Intelligence Scale for Children 4^th^ Edition; STRS: Student-Teacher Relationship Scale.

## Competing interests

PH’s institution has received payment from Eli Lilly and Janssen for consultancies; Eli Lilly, Janssen, Novartis and Shire for advisory boards; Eli Lilly, Janssen, Pfizer and Shire for speaker’s bureau; Eli Lilly and Celltech for the conduct of clinical trials. ES, DE, ES VA, BJ, OU and JN have no conflicts to declare.

## Authors’ contributions

ES, DE, VA, PH, BJ, OU, and JN contributed to the overall design and conception of the study and assisted with the writing of the grant application and revised this manuscript. ES, EJS and JN drafted this manuscript. EJS and ES contributed to study implementation. OU contributed to the statistical design of the study. All authors read and approved the final manuscript.

## Pre-publication history

The pre-publication history for this paper can be accessed here:

http://www.biomedcentral.com/1471-244X/13/18/prepub
